# Influence of elastic lumbar support belts on trunk muscle function in patients with non-specific acute lumbar back pain

**DOI:** 10.1371/journal.pone.0211042

**Published:** 2019-01-24

**Authors:** Christoph Anders, Agnes Hübner

**Affiliations:** Clinic for Trauma, Hand and Reconstructive Surgery, Division of Motor Research, Pathophysiology and Biomechanics, Jena University Hospital, Jena, Germany; Hochschule Trier, GERMANY

## Abstract

**Background:**

A well-known supportive treatment for acute nonspecific back pain, elastic back support belts, are valued for their ability to accelerate natural self-healing, but there are concerns of a deconditioning effect due to their reliance on passive stabilization.

**Methods:**

To evaluate the systematic effects of elastic abdominal belts on the trunk musculature, a total of 36 persons with acute lumbar back pain (no longer than one week) were divided into two groups: an abdominal belt wearing group (B) and a non-abdominal belt wearing control group (C). All were examined over a period of three weeks at three time points: T1 just after assignment, T2 one week later, and T3 further two weeks later. Surface EMG (sEMG) was used to record trunk muscle activation when walking on a treadmill at walking speeds of 2, 3, 4, 5, and 6 km/h. Similarly, pain intensity (VAS) and functional impairment (ODI) over time were recorded in both groups.

**Results:**

Over the observation period, a slight advantage for decreased pain intensity (C: p<0.05 T2 vs. T1; B: p<0.01 T2 vs. T1, p<0.05 T3 vs. T1) and decreased functional impairment (Cohen's d vs. T1, C: T2 0.45, T3 0.86; B: T2 1.1, T3 1.0) was observed for the belt group. For the belt group both oblique abdominal muscles exhibited significantly lower sEMG throughout the observation period (external abdominal oblique muscle: (T1), T2, (T3), internal abdominal oblique muscle: T1, (T2), (T3)) and the sEMG for the back muscles ranged from unchanged to slightly elevated for this group, but never reached statistical significance.

**Discussion:**

The reduced abdominal amplitude levels in the belt group likely result from the permanent elastic stabilization provided by the belt: the required elevated intra-abdominal pressure to enhance spinal stability is then provided by lessened abdominal muscle activity complemented by the belt’s elastic support. With regard to the back muscles, the belt, due to its movement-restricting effect, tends to activate the paravertebral musculature. In this respect, the effect of elastic abdominal belts on the trunk muscles is not uniform. Therefore, the present results suggest that the effect of elastic abdominal belts appears to be more of a temporary neutral alteration of trunk muscle coordination, with some trunk muscles becoming more active and others less, and not a case of uniform deconditioning as is suspected.

## Introduction

In addition to their common use in preventing back injury, e.g. during heavy load carrying in industrial settings, elastic lumbar support belts are often applied as a therapeutic device to accelerate the relief of suffering from acute back pain, one of the most common and expensive diseases in Germany [1]. But there are concerns in the field of back pain management that back support belts of all types in general might not be advisable because they bear the risk of deconditioning the trunk muscles and are at best neutral and at worst represent a hindrance rather than a help. This is reflected in the German national disease guidelines for back pain that do not recommend lumbar support belts [2]. This opinion is based on the idea that lumbar support belt’s supportive function partly replaces the necessary stabilizing function of trunk muscles [3, 4] which is equated with a threat of muscle deconditioning due to load removal [5]. However, this fear is in contrast to the proven helpful assistance of specifically, elastic lumbar support belts, in the treatment of acute back pain [6, 7], and the lack of evidence of any systematic deconditioning effect of lumbar support belts: Two recently published reviews found no evidence of reduced maximum strength or endurance [8] nor could they find consistent changes in trunk muscle activity [9]. However, both reviews rated their database quality as rather low [8] or largely heterogeneous [9], therefore requiring further investigations.

Despite intensive investigation of the efficacy of elastic lumbar support belts as an aspect of relief of acute back pain questions remain because causes of low back pain are not directly visible using typical physiological diagnostic techniques. The bulk of this research has relied on somewhat subjective data such as missed work days, compensation claims, and questionnaires obtained from acute back pain patients, regarding pain relief, functional restoration, and return to work [6]. As a result prior reviews conflict with one another [10–12].

One way of measuring the physiological effects of elastic back support belts in the treatment of non-specific acute low back pain is to make inferences based on changes in muscle function. Only a few such physiological studies on changes in trunk muscle function have been published as yet. These studies as a group utilized different methodologies and as yet have not provided a consensus [13]. There is need for more studies with similar methodologies to help answer what changes occur in muscle function from the application of elastic lumbar support belts in acute low back pain.

Own investigations in healthy subjects already provided hints that argue against the suspected deconditioning effect of elastic lumbar support belts [14], which is useful in trying to understand their use in prevention, but these results are not transferrable to therapeutic use in acute back pain patients. Therefore, the logical next step for us was to apply the same study measurement methodology to follow a cohort of patients suffering from non-specific acute back pain, thus combining surface EMG (sEMG) of muscle activation, with use of other important measures such as pain and functional disability. In detail we asked whether systematic differences in trunk muscle activity during treadmill walking over a 3-week follow-up period could be detected comparing patients with and without wearing an elastic lumbar support belt and whether changes over time in the intensity of pain or the extent of functional impairment would differ.

According to own previous results on the effects of elastic lumbar support belts in healthy volunteers during walking [14] we hypothesized to see initially reduced sEMG amplitudes for all trunk muscles while wearing elastic lumbar support belts, followed by a return to the values of non-wearers in the sequel for the back muscles while those of the abdominal muscles were expected to remain reduced. With respect to pain intensity and functional disability we expected a general improvement in both groups but a more distinct pain relief and improved functional status in the belt group.

## Methods

### Study design

The study was conducted as a comparative observational study over a three-week period. The study was approved by the Ethics Committee of the Friedrich-Schiller-University Jena (4205–09 / 14). The patients were recruited via press calls, referrals from treating physicians as well as posters.

### Study population criteria

Study participants included patients of both sexes between the ages of 30 and 60, who, at the time of recruitment had acute local back pain in the area between the iliac crest and the costal arch lasting no longer than one week. Exclusion criteria included permanent, or more than a week lasting pain, a BMI > 30, past surgery of the spine, the inability to implement the experiment mentally and/or physically (including a thorough check of ability to habituate to treadmill locomotion) as well as radiating pain in the legs or pain outside the defined region. Recurrent low back pain less than three times per year did not lead to exclusion from the study if the last pain episode was more than 3 months prior.

### Study participants

In the period 02/2015–04/2016, 284 potential study participants were contacted via the mentioned recruiting channels. Due to the inclusion and exclusion criteria, 38 persons could be included in the study. The most common reasons for exclusion from the study were longer-lasting or chronic low back pain and nonspecific pain sites. Furthermore, a total of two participants due to illness-related drop-out (one female and one male, both assigned to the control group) had to be excluded from the analysis.

After admission to the study subjects were assigned to the group belt (B) or control (C) in an alternating fashion. This was done separately for both sexes, with the initial assignment made to the C group. The composition of the two groups is shown in Table 1.

**Table 1:** Anthropometric characteristics of the initially admitted and analyzed study participants.

Initially admitted	BMI [kg/m^2^]	Height [cm]	Weight [kg]	Age [years]
Female C (n = 7)	23.8 ± 2.1	**164.4 ± 7.0**	**64.7 ± 10.5**	42.9 ± 17.6
Female B (n = 6)	23.5 ± 1.5	**164.5 ± 1.9**	**63.7 ± 4.7**	42.7 ± 9.9
Male C (n = 13)	24.5 ± 2.7	**181.8 ± 6.7**	**81.4 ± 12.0**	44.1 ± 15.3
Male B (n = 12)	25.3 ± 2.3	**179.0 ± 6.3**	**82.2 ± 9.7**	37.8 ± 9.3
Analyzed				
Female C (n = 6)	24.2 ± 1.9	**165.2 ± 7.4**	**66.3 ± 10.4**	45.8 ± 17.3
Female B (n = 6)	23.5 ± 1.5	**164.5 ± 1.9**	**63.7 ± 4.7**	42.7 ± 9.9
Male C (n = 12)	24.6 ± 2.8	**182.3 ± 6.7**	**82.1 ± 12.3**	45.8 ± 14.8
Male B (n = 12)	25.3 ± 2.3	**179.0 ± 6.3**	**82.2 ± 9.7**	37.8 ± 9.3

The numerical values are shown in bold if there were significant differences (p <0.05) between the women and men of the respective study group (C: control group (no belt), B: belt group).

In agreement with the sex-specificity of human anthropometric characteristics, study participants differed between sexes for height and weight [15, 16], but not for age and BMI values. Between groups C and B no systematic differences could be found.

### Investigation procedure

At the beginning of the first investigation time participants were habituated to the treadmill locomotion. Only when a natural gait pattern (clear view forward, normal arm swing, swinging legs) was achieved was the actual measurement preparation and examination begun. After successful treadmill habituation participants were instrumented (see Data acquisition below, duration about 20 minutes). The examination was carried out by asking patients to walk at speeds of 2–6 km/h in a 1 km/h gradation on a treadmill. Per walking speed always at least 40 strides were completed. The sequence of walking speeds was individually randomized (shuffling and blind selection between five cards indicating the particular walking speed) and then maintained for that participant for each of the three study sessions (Trials).

The Trials were performed at three time points: immediately after assignment or contact (T1), one week later (T2) and two further weeks later (T3). For the group with belt, T1 involved two scenarios: a complete examination without the elastic lumbar support belt (T1-1) and after a five minutes rest time another complete examination with the elastic lumbar support belt on (T1-2). For the investigation, the abdominal elastic lumbar support belt Lumbotrain (Bauerfeind AG) was used in the respective size (seven sizes, covering an abdominal girth range between 70 cm and 145 cm, compression class II) and gender-specific design. Its ventral and dorsal height was 17 cm and 23 cm (female sizes 1–2: 24 cm, female sizes 3–7: 27 cm), respectively. It comes with a triangularly shaped reinforcement element that is placed at the middle of the back with its downwards pointing angle over the tailbone. The Lumbotrain can be fastened in the front by pulling finger pockets evenly forward and fixing both sides in place with Velcro fasteners. The belt is made of a bi-elastic knit that stretches in horizontal and vertical directions and can be worn directly on the skin and also over a thin shirt. Patients were asked to wear the elastic lumbar support belt daily for a minimum of 4 hours, as recommended by the manufacturer. Wearing of the support is recommended during any kind of physical activity, but subjects were instructed to remove it during prolonged times of inactivity, i.e. during sitting or lying down. By this the German guidelines for the treatment of low back pain that among other things recommend physical activity were implicitly considered [2]. Daily wearing time and pain were documented in a pain diary, thus providing control of adequate compliance that needed to be met for all included study participants. Due to illness requiring bed rest, two persons dropped out from the study.

### Data acquisition

At each examination, participants’ current pain intensity was requested before and after the treadmill testing (VAS 1–10). After completion of the treadmill testing, the degree of functional impairment was assessed using the Oswestry Disability Inventory (ODI).

As a physiological measure, the surface EMG (sEMG) of six superficial trunk muscles was measured simultaneously from both body sides. The electrodes (H93SG, Covidien) were positioned according to the international recommendations [17, 18] and always by the same experienced investigator (CA). The positions used are shown in Table 2.

**Table 2:** Measured muscles and electrode positions.

Muscle	Electrode position and orientation
Rectus abdominis muscle (RA)	4cm lateral of navel, lower electrode at navel level, vertical
Internal abdominal oblique muscle (OI)	Along horizontal line between both ASIS's, distal electrode medial from inguinal ligament
External abdominal oblique muscle (OE)	Upper electrode directly below most inferior point of costal margin, on line to opposite pubic tubercle
Multifidus muscle (MF)	1 cm medial from line between PSIS's and 1st palpable spinous process, lower electrode at L4 level, parallel to line
Iliocostalis muscle (ICO)	Center between electrodes 1 cm medial of line between PSIS and the most inferior point of costal margin, L2 level
Longissimus muscle (LO)	Vertical, over palpable bulge of muscle (approx. 3 cm lateral midline) caudal electrode at L1 level

ASIS: anterior superior iliac spine. PSIS: posterior superior iliac spine.

In addition, one electrode pair was positioned along the heart axis to allow the elimination of QRS complexes through registration of cardiac activity. To detect the individual steps, pressure sensors were fastened in the heel area of the shoes. Secure seating of all electrodes was monitored throughout the entire examination and loose electrodes were replaced.

The signals were amplified (gain: 1000, Biovision), digitized (Tower of Measurement, sampling rate: 2000/s, anti-aliasing filter at 1000 Hz, resolution: 24 bit (0.596 nV/bit), DeMeTec) and stored on hard disk for further processing (ATISArec, GJB).

For the data analysis, the sEMG signals were band pass filtered between 20 Hz and 300 Hz and a 50 Hz notch filter was used to eliminate interferences from the power grid. The inevitable heart activity disruptions were individually and separately eliminated for each sEMG channel using a template-based algorithm [19]. Based on the pressure signals, heel contact times were detected. To ensure steady-state measurement conditions, only complete strides that did not deviate more than 10% from the respectively determined median stride time were used for further analyses. The sEMG signals were quantified as root mean square (rms) and smoothed with a 50 ms sliding averaging window. The valid strides were time normalized to 100% and quantified with a time resolution of 0.5% (201 measurement points). All data were subjected to a visual plausibility check and individual strides whose rms curves deviated more than 2 SD from the averaged curve were excluded from further analysis. From the remaining individual strides, averaged amplitude curves were calculated, which were then used in the analysis.

### Outcome parameters

For the analysis, the sEMG data, the scores in the ODI, and the details of the pain intensity before and after the treadmill testing were used. From the amplitude curves, the mean (time independent) SEMG amplitude levels were calculated.

The values for the pain and the ODI data were subjected to an ANOVA which took into account the trial numbers (T1 / T2 / T3, 3 levels) as well as the application of the elastic lumbar support belt (without/ with) as the between subject factor.

All sEMG data were covariance analyzed to identify the possible main effects "belt" (without / with, 2 levels), "Trial" (T1 / T2 / T3, 3 levels), "walking speed" (2/3/4/5/6 km / h, 5 levels), as well as "gender" (female / male, 2 levels) and their interactions.

All parameters were further statistically tested both in the course and between the two groups at all investigation times. In order to address the multiple test problem, i.e. to perform a correct statistical testing of the individual values, the accumulation of the type I statistical error had to be considered, which required a correction of the significance level [20]. However, with the available group size, the required level of significance was often not achievable. Therefore, the comparative consideration was additionally realized by the application of the effect size (ES, ANOVA, ANCOVA: partial Eta^2^ (η^2^_p_), η^2^_p_ ≥ 0.06: mean effect, η^2^_p_ ≥ 0.14: large effect [21]; single tests Cohen's d, d ≥ 0.4: average effect, d ≥ 0.8: large effect [22]).

## Results

### Pain

In the ANOVA, a general effect of the Trial (p = 0.048, η^2^_p_ = 0.189) could be demonstrated, but it was not possible to detect a systematic effect of the main effect "belt" (p = 0.210, η^2^_p_ = 0.052).

**Fig 1:**
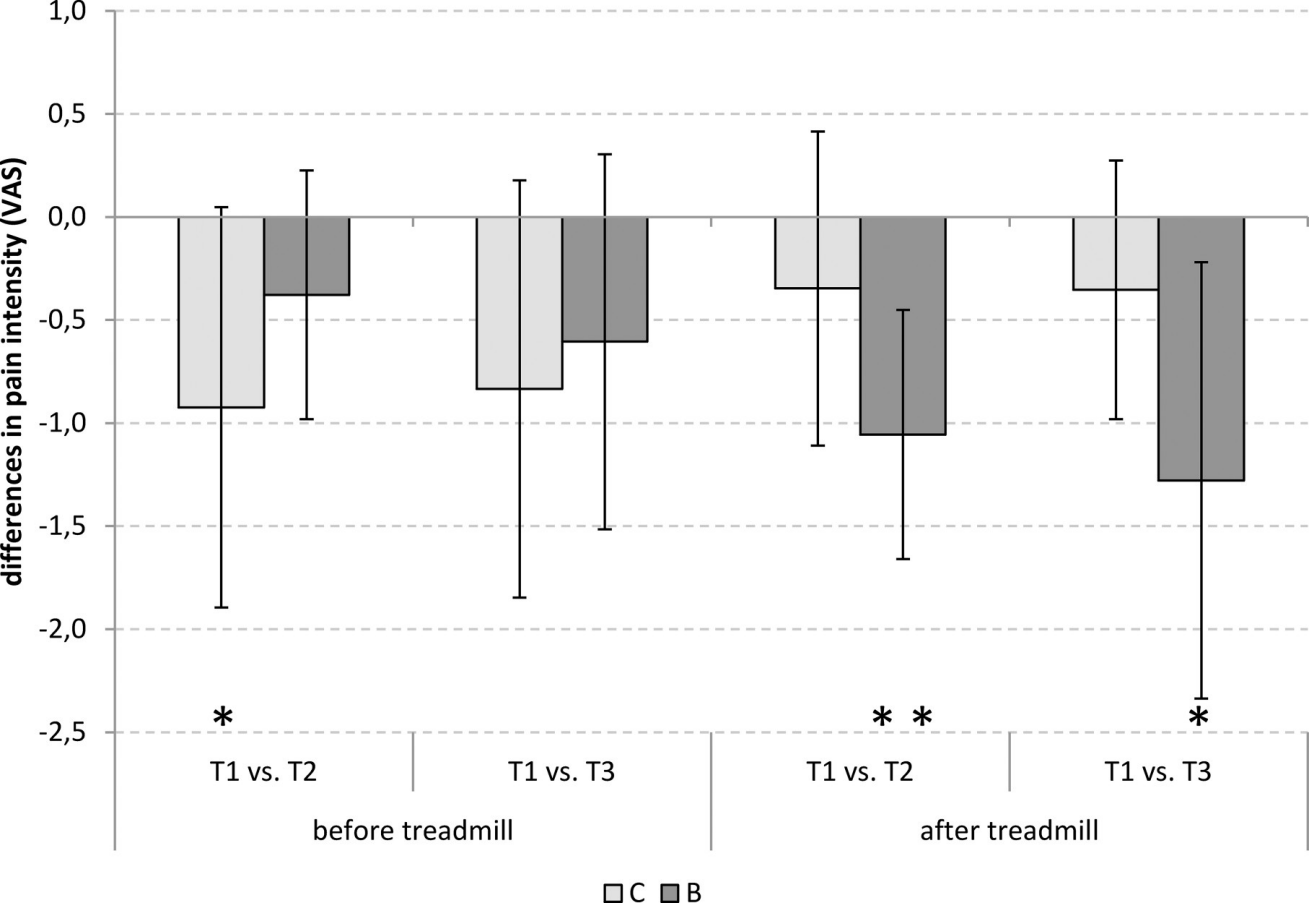
Differences in pain intensity at T2 and T3 in comparison with T1. C: control group (no belt), B: belt group. *: p < 0.05, **: p < 0.01.

For the individual comparisons, at no time group differences in pain intensity could be proven. At T1 the mean level of pain can be classified as comparatively low, showing pain levels of 1.95 ± 1.83 (B) and 2.54 ± 1.88 (C). However, the belt group subsequently showed a more marked reduction in the pain level compared to the baseline level (Fig 1), which was consistently significant compared to T1 at the end of the observation period.

### Functional impairment

For the values in the ODI also no systematic differences between the groups could be proven. Likewise, the values (see Table 3) indicate that the trend towards lower levels of impairment was observed throughout the observation period. Compared to T1, a significant reduction in functional impairment was demonstrated in all follow-up examinations for both groups. The effect sizes always showed higher values for group B.

**Table 3:** Top: Values (Mean ± SD) for the Oswestry Disability Inventory (ODI) for all observation times (T1 to T3). Bottom: Relative change compared to T1 (Mean ± SD). C: control group (no belt), B: belt group. The indicated effect sizes (ES, Cohen's d) are valid for both values at times T2 and T3 in comparison with T1.

		T1	T2	T3
C	ODI	22.5 ± 11.9	17.3 ± 10.8	13.9 ± 7.6
	ES vs. T1		0.450	0.859
B	ODI	24.8 ± 9.7	14.6 ± 9.1	13.8 ± 11.2
	ES vs. T1		1.083	1.049
			**T1 vs. T2**	**T1 vs. T3**
C	rel. Change [%]		-23.3 ± 31.0	-36.0 ± 21.9
B	rel. Change [%]		-37.0 ± 37.5	-41.7 ± 44.4

### SEMG amplitudes

In the ANOVA neither systematic effects of body side (p: 0.119–0.990) nor gender (p: 0.087–0.906) could be determined. The only exception to this was the data of OE, which showed a gender dependency (p: 0.007–0.062), independent of trial number (p: 0.209–0.923) or group affiliation to the belt or control group (p: 0.280–0.858). Therefore, the individual tests regarding existing differences between the Trials and the group differences were generally calculated with pooled data of both sexes and exemplarily with the values of the left side of the body.

### Mean amplitude values

**Fig 2:**
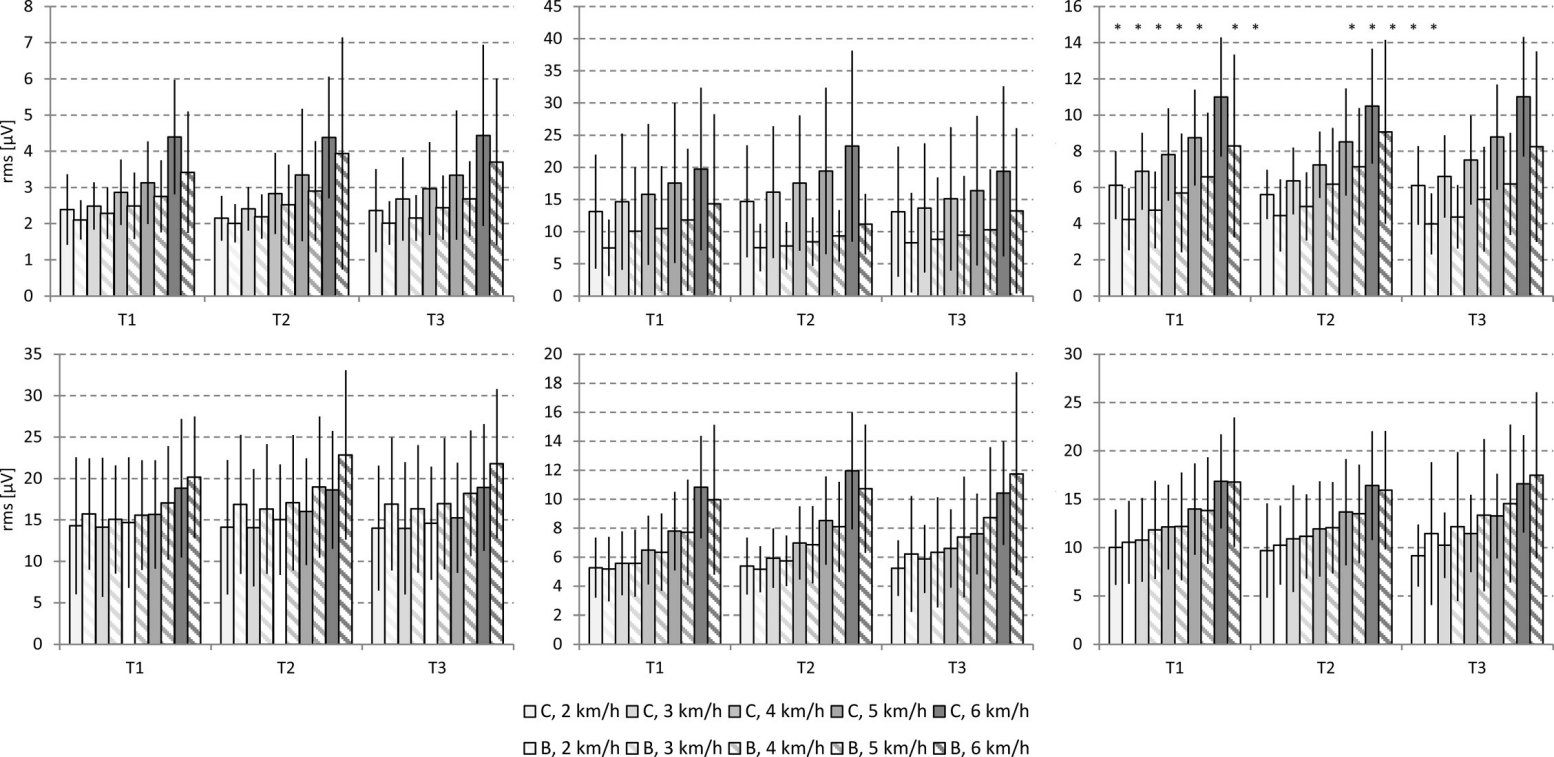
Mean SEMG amplitude (MW ± SD) values for the examined trunk muscles at T1, T2, and T3. Control group (no belt, C): filled columns, belt group (B): hatched columns. RA: rectus abdominis muscle, OI: internal abdominal oblique muscle, OE: external abdominal oblique muscle, MF: multifidus muscle, ICO: iliocostalis muscle, LO: longissimus muscle. The asterisks indicate significant differences (p<0.05) between the control and belt group.

With increasing walking speed an increase of mean amplitude values could be observed for all investigated trunk muscles. The RA showed the lowest levels with maximum mean amplitude values of less than 5 μV (T3, 6 km/h, Group C), while the OI showed the highest values of about 24 μV (T2, 6 km/h, Group C; see Fig 2).

Mean amplitudes of all abdominal muscles were lower in the belt group. This reached significance for both oblique abdominal muscles (p<0.05, OI: (T1), T2, T3, OE: T1 (T2), T3, see Fig 2). In contrast, mean amplitudes of back muscles showed no systematic differences between the C and B group.

### Grand averaged amplitude curves

**Fig 3:**
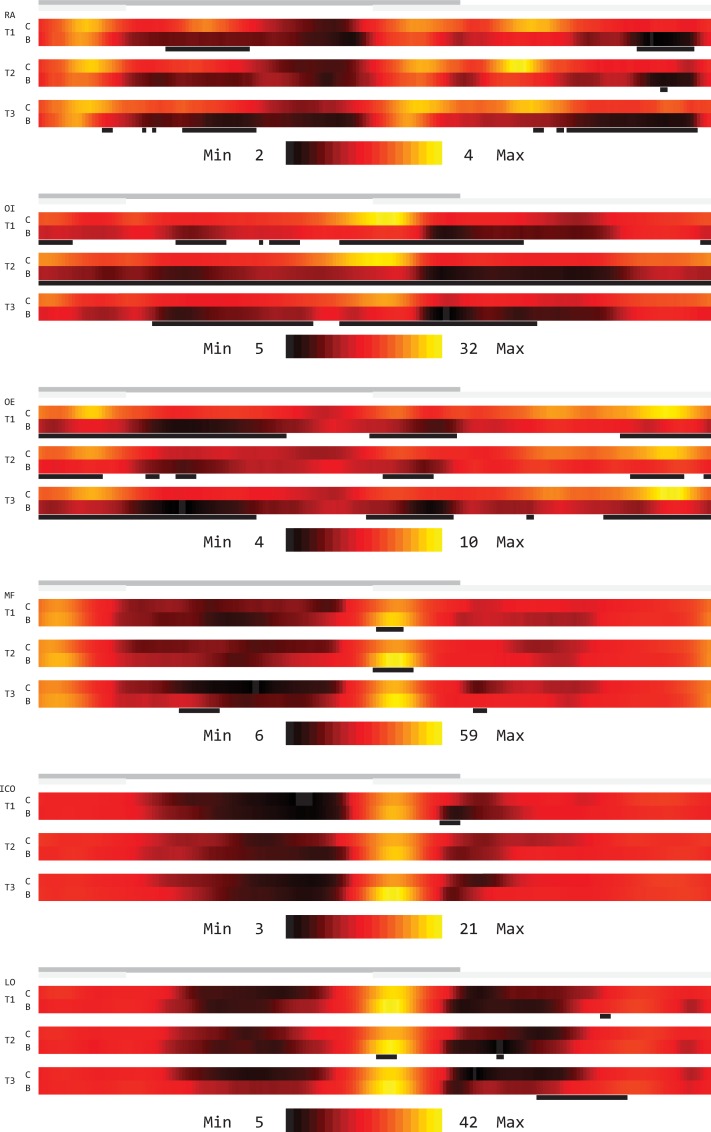
Color-coded representation of the grand averaged amplitude curves when walking at 4 km/h on the treadmill. The gray bars above the color-coded amplitude curves mark ipsilateral (dark gray) and contralateral (light gray) stance phases. The black bars mark differences between control group (C) and belt group (B) with an effect size of ≥ 0.5. RA: rectus abdominis muscle, OI: internal abdominal oblique muscle, OE: external abdominal oblique muscle, MF: multifidus muscle, ICO: iliocostalis muscle, LO: longissimus muscle.

The results for the grand averaged amplitude curves confirm the data of the mean amplitude values (see Fig 3): for the investigated abdominal muscles, significantly reduced amplitude values in the belt group compared to the control group could be detected (OI at T2). However, a systematic dependence on the trial number was not obvious. In the belt group for the examined back muscles, there were increased peak amplitude values at the respective heel strike time points, these achieved relevance for MF and LO several times. Furthermore, there were tendencies towards increased amplitude values for the MF and LO during the low-amplitude stance and swing phases, which again reached relevance at T3.

## Discussion

In the presented study, it could be shown that for patients with acute back pain over a three week period a noticeable reduction of back pain, and improved restoration of functional disability could be observed. These improvements were slightly augmented by wearing an elastic lumbar support belt. As for objective measures, systematic effects on the activity characteristics of trunk muscles during walking were observed using sEMG. While the subjective positive effects on pain reduction and functional impairment have already been described in the literature [6, 7], sEMG measurement of trunk muscles during everyday activities has not been described so far: For walking we found relevant and significantly reduced abdominal muscle amplitude levels, together with unchanged or increased amplitude values for the examined back muscles in the belt group. The observed changes for the abdominal muscles were evenly distributed over the entire stride. The changes for the back muscles, however, occurred only during phases of particularly high or low amplitude values. Thus, the effect of elastic lumbar belts on the trunk muscles is not generalizable and subject to muscle specific features.

### Subjective assessment levels: Pain & functional impairment

Pain intensity at study enrollment was lower than in other studies [6, 23], showing values of 1.95 ± 1.83 (B) and 2.54 ± 1.88 (C). Both the tendency to spontaneous improvement of acute low back pain, together with the already described improvement of clinical symptoms by wearing abdominal elastic lumbar support belts, were demonstrated in the current study [6]. The low initial pain level would potentially reduce clear systematic differences between both groups. Thus, with regard to accelerated and/or improved pain reduction, the known results were successfully re-evaluated.

The initial values for the functional disability (ODI) with 22.5 (C) and 24.8 (B) were also lower than described in the literature [24–29] but are in accordance with the comparatively low pain levels of the investigated cohort. Nevertheless, the expected reduction for the ODI of at least 4 points [28, 29], or 15% [24] from baseline level was significantly exceeded in both groups. In this respect, the data confirm, even in conjunction with the change in pain levels, the tendency of improved functional impairment in acute back pain, which, however, was significantly enhanced by wearing the elastic lumbar support belt. Interestingly, the reduced pain for the belt group could only be proven after the treadmill tasks, arguing for an activity-related effect of the elastic support belt. This particular result supports the idea of belt use during walking, whereby the spine is stabilized by increased intra-abdominal pressure (IAP, [25]). Other positive contributions of belt wearing would require further studies.

### SEMG-amplitudes

The differentiated effect of the elastic lumbar support belt on the amplitude levels already described in the summary of the results, at first sight appears contradictory to the outcome of improved symptomology. Closer analysis of the passive stabilizing properties of the elastic lumbar support belt suggests that the function of the abdominal muscles in acute back pain plays a key role: previous studies on back pain patients have demonstrated compensatory increases in activity and co-contraction indices, especially for abdominal muscles [3, 4]. By this the IAP will be elevated [30], which, in turn is known to improve the stability of the lumbar spine without the need for simultaneous back muscle activation [31]. Therefore, any elevated IAP whether it is solely caused by muscular activity or with the help of an elastic support should improve the stability of the spine [25–27, 32]. However, if the required IAP increase is only provided by an increase in muscular activity the risk of muscular fatigue is no theoretical issue. In extreme cases it may lead to temporary drops in muscular performance, and thus to temporary losses of the required stabilization. Abdominal muscles compared to back muscles have a higher proportion of type II muscle fibers [33–35] and therefore are particularly susceptible to fatigue-related failure to contribute adequately to spinal stability due to lower power output [36]. Since the wearing of the elastic support belts had a positive effect on symptoms over a three week period of time, the observed lower amplitude values of the abdominal muscles in the elastic lumbar support belt group was a positive effect of passive support to increase the IAP and therefore stiffening the lumbar spine. By this, possible fatigue-related abdominal muscle fails were successfully prevented. It is interesting to note in this case that according to the functional assignment of the trunk muscles, the lowest amplitude values were found for the RA, i.e. a functionally mobilizing muscle [37], whereas both oblique abdominal muscles, as globally stabilizing muscles, had comparatively higher amplitude values that were significantly reduced by the elastic lumbar support belt.

In contrast, it was not possible to identify general, but only punctual differences in the amplitude curves for the back muscles, in which the belt group always had the higher amplitude values than the control group. These momentary differences were accompanied by non-significant, but slightly elevated mean amplitude levels at T2 for MF and T3 for all back muscles, and were consistent with biomechanical model calculations that could not identify one single muscle, but the well-coordinated action of all trunk muscles to improve spinal stability [38]. As individual activation patterns have to be expected across all patients, the found differences are all the more meaningful, since they point to phase-specific modified muscle activation patterns if wearing the belt. Greater inter- and intra-individual differences in back muscle activation likely related to improved, phase specific trunk muscle co-ordination during walking.

In this study we have not investigated tasks performed in other belt studies of healthy volunteers, such as manual lifting [39], squat lifting [40, 41], maximum force [42] or endurance capacity tests [8], as these should not be executed by acute back pain patients. These single observation studies were conflicting and ultimately not useful for comparison to ours. If however we compare these results with our own previous results in healthy subjects [14], where we compared non-belt and belt use during walking in the same subjects, we see similar reductions in abdominal muscle activation. Also, in the current study we observed consistent differences between C and B groups over the three Trials, both of these congruities argue for a repeatable effect on abdominal activation of the elastic support belt during walking. We can also say that effect of the belt does not lessen over a period of three weeks.

Comparing both our studies for the back muscles, acute back pain patients, in contrast to healthy subjects, show phase specific elevated amplitudes of their back muscles while wearing belts. Differences between acute low back pain patients and healthy subjects are not surprising because different intermuscular co-ordination is seen during acute back pain [4]. Different then for back muscles, the abdominal muscles show comparative effects of belt wearing in both patients and healthy subjects during walking.

### Conclusion

Thus, first of all, there was clearly no sign that wearing a lumbar compression elastic lumbar support belt leads to a general deconditioning of the back muscles. The amplitude levels of the abdominal muscles were significantly reduced in the belt group with no specific stride phase attribution, most probably caused by the passive elastic support of the belt. Over the observation period all patients showed a reduction of their pain level and improvement of their functional impairment that was slightly augmented in the belt group. The observation period of three weeks is consistent with the natural course of acute low back pain and the observed results demonstrated a repeatable effect of the belt. Future studies should be based on a broader database and involving other common activities like twisting, bending and light lifting, and eventually include patients with recurrent back pain.

## Limitations

For the study, only 36 people could be included in the analysis, which makes broad generalization from the results impossible. The 36 patients who were analyzed were selected from a total of 284 patients who responded to the recruitment effort, representing a 12.7% inclusion rate—higher than in other studies [23]. Also, the three-week post-study observation time was relatively short compared to other studies that continued for 12 months, to describe the natural course of acute low back pain [23]. On the other hand, the duration of observation was chosen on the basis of previous studies on the effectiveness of elastic lumbar support belts [6] and also based on the fact that after four weeks only small changes are to be expected [23].

## Supporting information

S1 Dataset All analysed data are stored in the zipped file "Dataset lumbar belt final.zip" that contains ODI scores, VAS scores, the time normalized sEMG data, and the anthropometric characteristics of all study participants. (ZIP)
